# Key Processes for *Cheirolophus* (Asteraceae) Diversification on Oceanic Islands Inferred from AFLP Data

**DOI:** 10.1371/journal.pone.0113207

**Published:** 2014-11-20

**Authors:** Daniel Vitales, Alfredo García-Fernández, Jaume Pellicer, Joan Vallès, Arnoldo Santos-Guerra, Robyn S. Cowan, Michael F. Fay, Oriane Hidalgo, Teresa Garnatje

**Affiliations:** 1 Laboratori de Botànica – Unitat associada CSIC, Facultat de Farmàcia, Universitat de Barcelona, Barcelona, Catalonia, Spain; 2 Institut Botànic de Barcelona (IBB-CSIC-ICUB), Barcelona, Catalonia, Spain; 3 Área de Biodiversidad y Conservación, Universidad Rey Juan Carlos, Móstoles, Madrid, Spain; 4 Jodrell Laboratory, Royal Botanic Gardens, Kew, Richmond, Surrey, United Kingdom; 5 Unidad de Botánica-ICIA, Puerto de la Cruz, Canary Islands, Spain; Instituto de Higiene e Medicina Tropical, Portugal

## Abstract

The radiation of the genus *Cheirolophus* (Asteraceae) in Macaronesia constitutes a spectacular case of rapid diversification on oceanic islands. Twenty species – nine of them included in the IUCN Red List of Threatened Species – have been described to date inhabiting the Madeiran and Canarian archipelagos. A previous phylogenetic study revealed that the diversification of *Cheirolophus* in Macaronesia started less than 2 Ma. As a result of such an explosive speciation process, limited phylogenetic resolution was reported, mainly due to the low variability of the employed molecular markers. In the present study, we used highly polymorphic AFLP markers to i) evaluate species' boundaries, ii) infer their evolutionary relationships and iii) investigate the patterns of genetic diversity in relation to the potential processes likely involved in the radiation of *Cheirolophus*. One hundred and seventy-two individuals representing all Macaronesian *Cheirolophus* species were analysed using 249 AFLP loci. Our results suggest that geographic isolation played an important role in this radiation process. This was likely driven by the combination of poor gene flow capacity and a good ability for sporadic long-distance colonisations. In addition, we also found some traces of introgression and incipient ecological adaptation, which could have further enhanced the extraordinary diversification of *Cheirolophus* in Macaronesia. Last, we hypothesize that current threat categories assigned to Macaronesian *Cheirolophus* species do not reflect their respective evolutionary relevance, so future evaluations of their conservation status should take into account the results presented here.

## Introduction

In the last two decades, the Macaronesian archipelagos (Canary Islands, Cape Verde, Azores, Madeira and Savages) have attracted much interest from researchers studying plant diversification and radiation processes [1]. These volcanic islands provide a wide variety of ecological conditions, geological ages and geographical isolation scales [2]–[4], which promote the existence of a mosaic of habitats that represent an excellent natural laboratory in which to study selection forces and evolutionary processes. The Macaronesian archipelagos have been recognized as a hotspot of plant diversity [5], and have rapidly become a popular model system for scientists to test many speciation hypotheses, both empirically and theoretically. The wide diversity of habitats found in Macaronesia – spanning from xerophytic coastal cliffs to subalpine belts – has served to demonstrate the role of adaptive radiation in a range of plant groups (e.g. *Argyranthemum* Webb, [6]; *Sonchus* L. alliance, [7]; *Aeonium* Webb & Berthel., [8]; *Echium* L., [9]; *Tolpis* Adans., [10]). Indeed, niche pre-emption through adaptive radiation is the most prevailing hypothesis to explain the high degree of endemism and monophyly within Macaronesian lineages [11], [12]. Furthermore, during the geological history of Macaronesian archipelagos, several islands have emerged, disappeared and/or changed their relative geographical position (see [4]), promoting complex isolation-connection and colonisation processes between islands and between islands and the continent. This complexity in volcanic archipelagos has given rise to numerous study cases examining the relative importance of vicariance versus dispersal in shaping insular biotas [13]–[15]; the role of islands as regions from which taxa might colonise continents and other archipelagos [16], [17] and the different stages of colonisation and radiation processes in relation to the ontogeny phases of oceanic islands [18], [19].

Occasionally, island radiations occur over a short period of time, resulting in ecologically and morphologically distinct taxa, but leading to poor molecular differentiation [9], [20]–[23]. These cases, in which explosive species radiation takes place are among the most interesting and least understood evolutionary events, maybe due to the difficulty in carrying out species level analyses [24]–[26]. Rapid island radiations have been generally associated to some features – such as small population size, release from previous ecological constraints, and adaptation to new niches – recurrently observed in a wide variety of species and island archipelagos [27]. However, since traditional phylogenetic studies usually provide little resolution in delimiting taxonomical boundaries and untangling the relationships among these rapidly evolving species, the precise role of morphological, life history, and physiological traits and their genetic basis in explosive plant radiations remain essentially unsolved [28].

**Figure 1:**
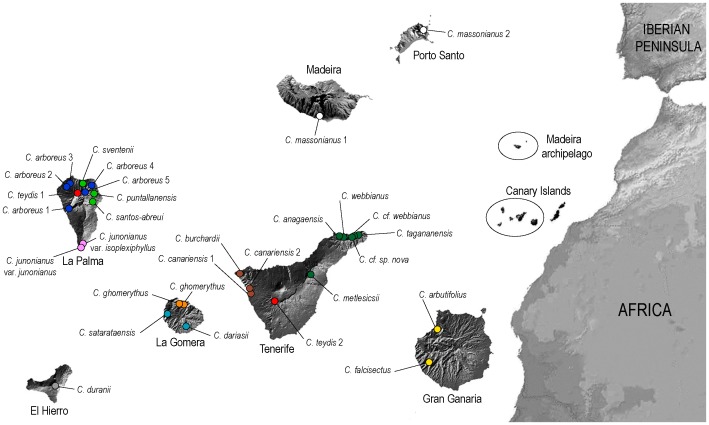
Geographic location of the 29 sampled populations of Macaronesian *Cheirolophus* species. Colour coding circles correspond to genetic structure derived from Bayesian mixture analysis of AFLP markers implemented in BAPS.

The Macaronesian *Cheirolophus* Cass. (Asteraceae) complex comprises 20 species (out of the 30 constituting the whole genus), with *Cheirolophus massonianus* (Lowe) A.Hansen & Sunding occurring in the Madeiran archipelago, and the remaining species distributed across the western Canary Islands (see Fig. 1). Although they are usually associated with humid basalt cliffs, a few taxa have adapted to inhabit very diverse ecological zones of the archipelago [29], [30]. Most of the island *Cheirolophus* are subshrubs, shrubs or even arborescent shrubs, showing a clear increase in woodiness relative to their continental congeners and a general shift towards inflorescences with white to purple flowers arranged in a candelabrum-like pattern. Some species are relatively widespread throughout a single island (e.g. *C. webbianus* (Sch.Bip.) Holub from Tenerife), whereas others are found on two different islands (e.g. *C. teydis* (C.Sm.) G.López from Tenerife and La Palma and *C. massonianus* from Madeira and Porto Santo). However, most species are narrow endemics occurring in a small number of restricted localities with only a few hundred or less individuals. Consequently, nine Macaronesian species have been included in the IUCN Red List of Threatened Species [31] as vulnerable, endangered or critically endangered. In addition, 17 of the species or subspecies endemic to the Canary Islands are included in the 2010 Red List of Spanish Vascular Flora [32]. In those cases where many related taxa are under threat and conservation strategies must be prioritized, taxonomic knowledge becomes of special relevance and efforts should be made to establish accurately the boundaries of the species concept. To achieve this goal, the recent “unified species concept” advocates the use of diverse lines of evidence (e.g. monophyly at one or multiple DNA loci, morphological diagnosis, ecological distinctiveness, etc.) so that a higher degree of corroboration in taxonomic delimitation is attained [33]. In recent times, conservation genetics has become an essential approach for evaluating the status and the level of threat at the species and population levels, identifying the genetic basis of processes which may potentially lead into an extinction vortex and informing authorities about management priorities for endangered species and/or populations [34].

Several investigations based on morphological, cytogenetic, isozymes and DNA sequencing data have been previously used to infer evolutionary relationships and taxonomy for the 29 currently recognized species of *Cheirolophus* [35]–[40]. These studies revealed that the genus arose in the Mediterranean region with subsequent dispersal towards Macaronesia, but they failed to reconstruct the relationships among insular congeners. A recent phylogenetic study featuring relaxed-clock dating analyses and diversification tests (involving sequences of various nuclear and plastid regions and sampling several populations per species, [41]) has evidenced a recent origin of Macaronesian *Cheirolophus* radiation. The age of this group was estimated to be 1.74 Ma (95%HPD 0.82–2.93), implying a diversification rate of 0.34–2.84 species Myr^−1^. Indeed, this diversification rate is comparable to those exhibited by the Hawaiian *Bidens* L. (0.3–2.3 species Myr^−1^) or Macaronesian *Echium* (0.4–1.5 species Myr^−1^), considered as the fastest plant radiations on volcanic islands documented to date [22]. Bayesian phylogeographic and ancestral range analyses were also applied in the same study, highlighting the major role of allopatric speciation to explain current diversity in Macaronesian *Cheirolophus* [41]. Geographical isolation among populations and long distance dispersal (both intra and inter-island colonisations) were proposed as the main forces driving the rapid diversification of the group, but introgression and emerging ecological adaptation were also suggested as additional factors reinforcing the speciation process. Despite such efforts, the employed nrDNA and cpDNA markers provided very low variability within the Macaronesian species, thus hampering accurate inferences about the role played by those evolutionary mechanisms potentially involved in the radiation of *Cheirolophus*.

Amplified fragment length polymorphism (AFLP) analyses have been found to provide insights into interspecific relationships in diverse animal and plant groups when other methodologies have failed in this attempt (e.g. [42]–[49]). Indeed, several authors have suggested that this technique can be especially useful in reconstructing phylogenetic relationships among species that have diverged or radiated recently [50]–[53]. Although some considerations must be taken into account for its use, AFLP are currently widely employed in molecular ecology and evolution research [54]–[59]. In fact, this DNA fingerprinting approach has proven to be particularly suitable for evaluating the genetic structure of plant species in different oceanic archipelagos, and hence elucidating the potential evolutionary forces – such as gene flow or genetic drift – that influence the distribution of genetic diversity among individuals, populations, and species [60]–[62].

AFLP fingerprinting – complemented with morphological and other molecular data – was employed here with the main objective of unravelling the explosive radiation that *Cheirolophus* underwent in the Canarian and Madeiran archipelagos. We applied phylogenetic and population genetic approaches to (i) evaluate the taxonomic identity of the Macaronesian species and to (ii) disentangle the evolutionary relationships between them. Additionally, we studied the patterns of genetic diversity within and between populations to (iii) infer the role of the evolutionary processes potentially involved with this explosive radiation (i.e. geographic isolation, ecological adaptation and introgression).

## Materials and Methods

### Sampling strategy

One hundred and seventy-two individuals from 29 populations of 20 Macaronesian *Cheirolophus* species were sampled, covering the whole taxonomic diversity recognized for the Madeiran and Canarian archipelagos (see Fig. 1 and Table S1 for further geographical details of sampling sites). One to 12 individuals per population were included in the study depending on the material availability and the uneven success of DNA extraction and AFLP procedures. We decided to incorporate those populations containing extremely scarce sampling (one or two individuals) because of their distinctiveness: (1) it was the only material available for the taxon (*C. dariasii* (Svent.) Bramwell; *C. cf*. *webbianus*); (2) the inclusion of that particular population was essential to understand the distribution of the species (*C. teydis* from La Palma; *C. massonianus* from Porto Santo); or (3) the additional subpopulation completes the sampling of extremely local species (*C. metlesicsii* Parada). Leaf material for DNA extraction was collected from plants in the field, dried in silica-gel and stored at room temperature. Insular Cabildos of Tenerife, Gran Canaria, La Gomera, La Palma and El Hierro provided all the necessary permits to collect samples from protected natural areas in the respective islands. The Canarian Council of Education, University and Sustainability issued the authorization to collect samples of the protected species listed on Table S1. Samples used of the endangered *C. massonianus* were provided by the Botanical Garden of Madeira.

### DNA extraction, AFLP protocol and nrDNA sequencing

Genomic DNA was extracted from fragments of silica-gel dried leaf tissue following the protocol of Doyle and Doyle [63] with slight modifications. DNA samples were cleaned using QIAquick columns (Qiagen, Valencia, CA, USA) and their quality and DNA concentration were determined using NanoDrop ND-1000 spectrophotometry (ThermoScientific, Wilmington, DE, USA). The AFLP technique was carried out following the protocol described in Vos et al. [64] in accordance with the modified AFLP Plant Mapping Protocol (Applied Biosystems Inc. Foster City, CA, USA) using *Eco*RI and *Mse*I with 500 ng of isolated genomic DNA per sample. Eighteen primer pair combinations were tested on six individuals from three different populations to screen those producing the most informative and readable profiles. Three pairs of primers were selected giving the most polymorphic and scorable polymorphic pattern: EcoRI-CTT/MseI-AC; EcoRI-CTC/MseI-AA; and EcoRI-CAG/MseI-AT. The success of each step was tested by running the PCR products on a 1.5% agarose gel. Fragments were run on an ABI Prism 3100 Genetic Analyzer (Applied Biosystems Inc.) with 10 µL of High Dye (deionized formamide) and 0.2 µL of GeneScan 500 ROX Size Standard per sample. Amplified fragments were genotyped as present/absent using GeneMarker AFLP/Genotyping software (version 1.9; SoftGenetics, LLC., State College, PA, USA).

For an initial scoring, all alleles within a range of 50 to 490 bp were considered. Afterwards, visual correction was carried out to eliminate erroneous peaks (low intensity or no reproducibility). AFLP error rates were calculated following [65]. Twenty-five random samples per primer combination were replicated to ensure reproducibility, repeating all parts of the AFLP protocol (extraction, digestion, pre-selective and selective PCR). All alleles with an error rate >5% were eliminated. In order to test the occurrence of size homoplasy, we also calculated the correlation between AFLP fragment sizes and frequencies using AFLP-SURV 1.0 [66].

We also employed some nuclear ribosomal DNA sequences to examine the potential role of genetic introgression in the radiation of Macaronesian *Cheirolophus*. Only those populations (see Table S2) showing clear evidences of interspecific gene flow according to our AFLP data – together with some of their putative parental species – were studied. Sequencing procedures, GenBank accessions and any other information about the material and methods employed can be found in [41]

### AFLP genetic diversity and population differences

The use of AFLP data (dominant markers) for estimating allelic frequencies implies the consideration of an outcrossing mating system and near random mating. This means that those populations would be under Hardy-Weinberg equilibrium [67]. *Cheirolophus* has a predominantly outcrossing mating system and is pollinated by generalist insects, so one expects near random mating in the studied populations.

To estimate genetic diversity in each population showing more than one sampled individual, the following parameters were calculated: a) private alleles; b) rare alleles, present in <10% of the samples; and c) unbiased heterozygosity (Hj), calculated using TFPGA v.1.3 [68]. Further measures of genetic diversity were estimated through: (i) the frequency-down-weighted marker values (DW) index of [69] using AFLPDAT [70]; (ii) the band richness (Br), which is the number of phenotypes expected at each locus, and can be interpreted as an analogue of the allelic richness, ranging from 1 to 2 [71]; and (iii) the percentage of polymorphic loci (PLP) with a significance of 1% (P = 0.99). Br and PLP indices were calculated according to the rarefaction method of Hurlbert [72], and conditioned to the smallest population size (N = 3) allowed by the software AFLPDIV v.1.0 (http://www.pierroton.inra.fr/genetics/labo/Software/Aflpdiv/). As a consequence, Br and PLP were not calculated for populations having less than three individuals. To assess whether genetic diversity indexes (i.e. Hj, DW, Br and PLP) differed among islands, diversity range values were compared in a non-parametric Kruskal-Wallis test using package RCMDR [73] implemented in R software [74].

Pairwise *F*_ST_ values were estimated for each pair of populations included in this study (Table S1) using AFLP SURV v.1.0 [75]. Significance was evaluated through 10000 permutations.

### Phylogenetic analyses

To address questions of species delimitation and evolutionary relationships, AFLP data generated from the three selected primer pairs were combined into one large matrix and analysed together. Phylogenetic trees were reconstructed for this combined dataset using neighbor-joining (NJ) and Bayesian inference methods. NJ trees were built using Nei-Li distances [76] and 1000 replicates with NTSYS PC v.2.1 software [77]. The support for specific nodes for the NJ tree was calculated using bootstrap [78] with 10000 replicates. Bayesian inference was performed in MrBayes v.3.1.2 [79] using a F81-like model for restriction sites [80], [81] and running four independent chains each of length 10 million sampling every 100 trees. Convergence was assessed using Tracer v.1.4.1 [82] and a burn-in of 1 million trees was discarded. The remaining trees were used to construct a 50% majority rule consensus tree.

Additionally, non-tree-building approaches have been recommended to avoid conflicting phylogenetic signals in those cases in which the analyses of the data do not exhibit a bifurcating tree-like behaviour [83]. Specifically, network methods have been proposed to resolve the uncertainty of these processes [83], [84]. We used the Neighbor-Net method [85] carried out with SplitsTree v.4.10 [83] to construct a distance-based network for the AFLP dataset using the Jaccard coefficient [86], which is restricted to shared band presence rather than shared absence.

### Genetic structure: spatial patterns, Bayesian clustering and PCoA

We performed Mantel tests to evaluate the spatial effect in the genetic differences between populations using two similarity matrices. Genetic distance matrices – with and without *C. massonianus* from Madeira – were constructed with *F*_ST_ values between populations, and geographical matrices were calculated by the spatial distance (X and Y coordinates) between populations using ArcGIS v.9.1 (ESRI, Redlands, CA, USA). Mantel tests were performed with the vegan package [87] implemented in R software [74] using 100000 permutations and considering a *p*-value limit of 0.05. Because most populations were from the Canary Islands and only two of them are from Madeiran archipelago, distant from the remainder (>500 km), the Mantel test analysis was repeated without these populations.

Bayesian clustering analysis were carried out using STRUCTURE v.2.3 [88]. We applied the admixture ancestry model and the correlated allele frequencies. Ten independent simulations were run for each possible number of genetic groups (*K*) from *K* = 1 to 29, using a burn-in period of 10^5^ generations and run lengths of 5×10^5^. To estimate the number of genetic groups (*K*) we selected the *K* value that maximizes the probability of the data L(*K*). We also considered the criterion proposed by Evanno et al. [89] to estimate the best value of *K* for our data set, based on the rate of change in the probability between successive *K* values, Δ*K*. Bayesian analyses of the genetic structure were also conducted with BAPS (Bayesian Analysis of Population Structure, Spatial Clustering of Groups, [90]), which uses stochastic optimization instead of Markov chain Monte Carlo to find the optimal partition. We performed a mixture analysis of individuals with the geographic origin of the samples used as an informative prior (‘spatial clustering of individuals') or without this prior (‘clustering of individuals’). BAPS simulations were run with the maximal number of groups (*K*) set from 1 to 29. Each run was replicated 10 times, and the results were averaged according to the resultant likelihood scores. The output of the mixture analyses were used as input for population admixture analysis [90], with the default settings in order to detect admixture between clusters.

Some authors have warned about a degree of over-splitting in the genetic structure analysed with AFLP markers (e.g. [54]) and have suggested the use of simpler statistical analyses to compare the pattern found by Bayesian methods. Similarities among individuals were also studied via Principal Coordinate Analysis (PCoA, [91]) using the Jaccard distance, in order to detect other possible relations that could not be visualized with assignment methods or phylogenetic analyses. This procedure was carried out with R software [74] using the vegan package [87].

Finally, we conducted analysis of molecular variance (AMOVA; [92]) using ARLEQUIN v.3.5 [93] to estimate genetic differentiation following an alternative and widely used non-Bayesian approach that does not assume Hardy-Weinberg equilibrium or independence of markers. A first AMOVA analysis was implemented without taking regional structure into account. We also carried out four independent AMOVAs grouping the populations of *Cheirolophus* according to: i) their current taxonomic affiliation; ii) their island origin; iii) the most plausible model proposed by STRUCTURE; and, iv) the clustering proposed by BAPS. Pair-wise genetic distance between individuals using the square Euclidean distance was used for the AMOVA analyses, considering three levels: within populations, between populations within regions and between regions. Only two levels (within and between populations) were considered when no additional grouping structure was applied.

### Morphological revision and analyses

Diagnostic morphological characters classically used in taxonomical treatments of Macaronesian *Cheirolophus* were also analysed in this study. Morphological traits were measured from herbarium vouchers, cultivated individuals and/or gathered from the literature depending on the species. In the present study, morphological data was only considered for a given species when at least three different individuals where available for measurements. We obtained accurate data from 16 species for six morphological variables widely employed to distinguish among Canarian *Cheirolophus* taxa (Table S3) (including leaf length, leaf width, size of capitula, plant size, floral colour and leaf shape). Qualitative traits (i.e. floral colour and leaf shape) were coded numerically (+1 for whitish and −1 for rose-colored flowers; +1 for entire, −1 for divided and 0 for intermediate/both leaf shapes). All the variables were standardized by subtracting the character mean from each species measure and then dividing by the character standard deviation. The resulting matrix was analysed by Principal Components Analysis (PCA) using RCMDR [73] implemented in R software [74].

In order to correlate genetic AFLP data with morphological traits, we carried out Mantel tests and a generalized analysis of molecular variance (GAMOVA; [94]). Mantel tests were performed with R software [74] and the package vegan [87] computing 100000 permutations and considering a *p*-value limit of 0.05. Complementary to Mantel test, GAMOVA analyses provides a regression-based method of the analysis of molecular variance (AMOVA; [92]) that could be interpreted as a multiple regression. GAMOVA approach is an especially suited tool to identify, associate and evaluate the relationships between the differences in a phenotype or environmental variable of interest between some populations and the genetic variations among the same populations. The significance between genetic differentiation (i.e. *F*_ST_) and six morphological traits (see above) was tested by running a regression matrix with a permutation test with 10000 repetitions.

## Results

### AFLP genotyping and filtering

Initially, 371 alleles were obtained from automatic genotyping. After manual correction, error rates calculation, elimination of small and troublesome alleles and low intensity peaks, a final matrix with 249 (67.1%) alleles were considered for subsequent analyses. The final data set obtained showed an error rate of 3.2%, which is below the maximum error rate percentage accepted for good AFLP reproducibility (5%) [95].

### Population genetics: diversity and differentiation

Within-population genetic diversity measures are shown in Table S1. Private fragments were scarce across the studied populations; only three were detected, one each in *C. santos-abreui* A.Santos, *C. satarataensis* (Svent.) Holub and *C. tagananensis* (Svent.) Holub populations. The frequency of rare fragments was, conversely, much higher (10.6%), but most of these were shared by two or more populations. Hj diversity values were similar in all populations, ranging between Hj = 0.0459 (*C. duranii* (Burchard) Holub), and Hj = 0.1539 (*C. arboreus* (Webb & Berthel.) Holub from Los Tilos) in populations with more than three sampled individuals, with a mean value of Hj = 0.07892. The frequency-down-weighted marker values (DW) index showed overall high values, but considerable differences were observed among populations (ranging from 8.65 in *C. junonianus* (Svent.) Holub var. *isoplexiphyllus* to 19.75 in *C. webbianus*). The percentage of polymorphic loci (PLP %) ranged from 10.1% in *C. duranii* to 77.0% in *C. arboreus* (Los Tilos), and band richness (Br) ranged from 1.08 in *C. junonianus* var. *junonianus* to 1.46 in *C. arboreus* (Los Tilos). The DW was the only diversity index showing significant differences among islands (Kruskal-Wallis chi-squared = 15.02, df = 5, *p*-value<0.05); La Palma populations showed the lowest DW values. Pair-wise *F*_ST_ comparisons were significant (Table S4; mean value *F*_ST_±SD = 0.259±0.185 with all populations, mean value *F*_ST_±SD = 0.302±0.051 with populations with more than three individuals sampled). The biggest difference was found between *C. puntallanensis* A.Santos and *C. arbutifolius* (Svent.) G.Kunkel (*F*_ST_ = 0.577). The pair-wise comparisons between populations of the same species were much smaller than those between species (e.g. mean *F*_ST_ = 0.055 between *C. ghomerythus* (Svent.) Holub populations).

### Phylogenetic analyses of Macaronesian *Cheirolophus* based on AFLP data

**Figure 2:**
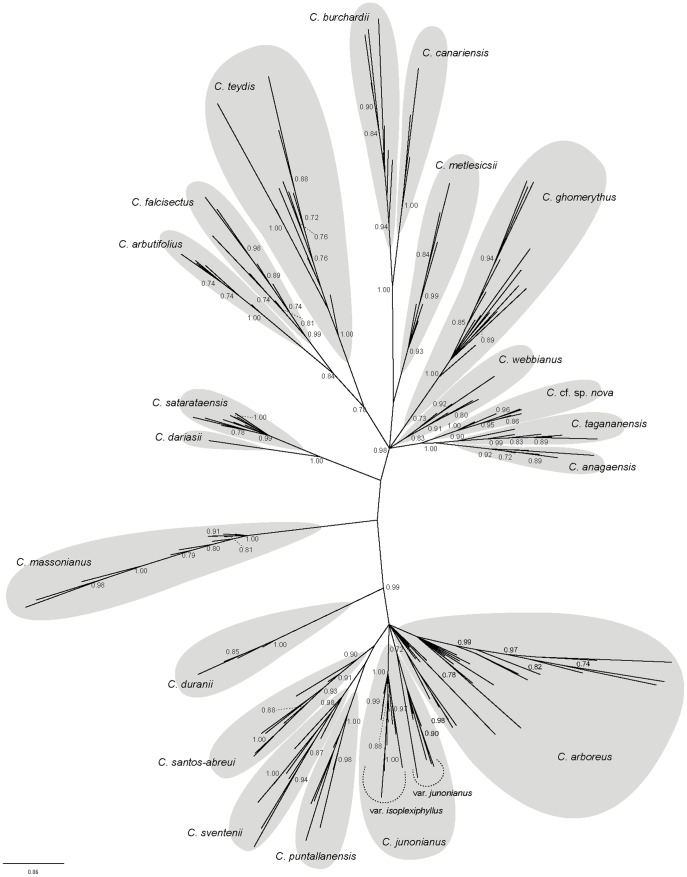
Phylogenetic analysis of AFLP data. Unrooted 50% majority rule tree from Bayesian analysis of the combined AFLP dataset for all Macaronesian species of *Cheirolophus*. Posterior probability values ≥ 70 are shown near each branch.

Trees constructed with the combined AFLP data using NJ and Bayesian estimation had generally similar topologies. Tree-building analyses of AFLP data resulted in similar fully-resolved assignment of individuals into species classically identified by diagnostic morphological and ecological characters (e.g. Bayesian 50% majority rule tree in Fig. 2; other trees not shown). However, species relationships were poorly resolved and only a few groups were partially identified. All trees included a large, strongly supported clade grouping most species from La Palma plus *C. duranii* from El Hierro (posterior probability, PP = 0.99; Fig. 2). Other supported smaller lineages grouped species from the Taganana peninsula (*C. tagananensis*, *C. anagaensis* A.Santos, *C. cf. webbianus* and *C. cf. sp. nova*; PP = 0.99) or species from the Teno mountains (*C. canariensis*, *C. burchardii* Susanna; PP = 1.00), in both cases from Tenerife. The phylogenetic analyses also confirmed the close relationship between *C. satarataensis* and *C. dariasii* (PP = 1.00), until recently considered as subspecies of the same species. The two populations of *C. teydis* from Tenerife and La Palma grouped together but were not embedded in a resolved clade. Similarly, *C. arbutifolius* and *C. falcisectus* Svent. ex Montelongo & Moraleda (both from Gran Canaria) appeared closely related (PP = 0.84) but their phylogenetic position in the genus was not fully resolved.

**Figure 3:**
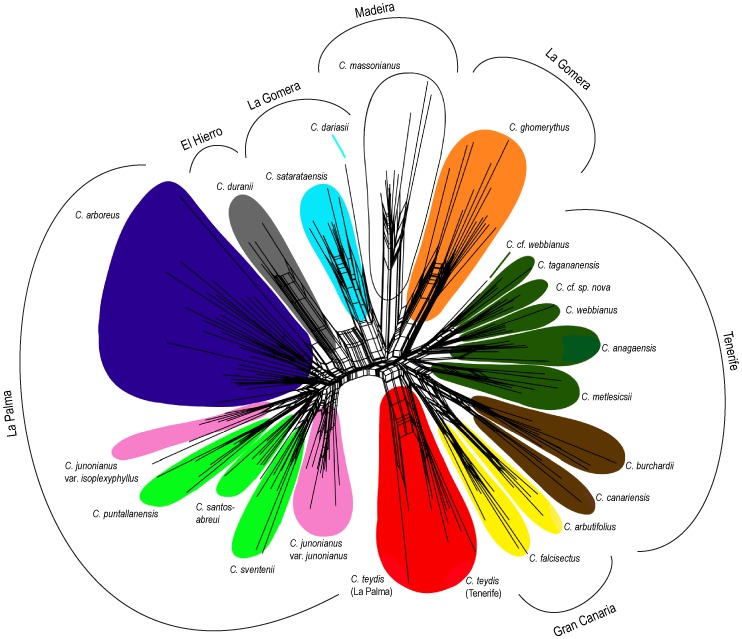
Neighbor-Net of AFLP data obtained from Macaronesian *Cheirolophus*. Colour coding profiles delimitate the different species indicating the genetic clusters assigned by BAPS.

The Neighbor-Net (NN) analysis, although indicating considerable reticulation in the data, resolved most of the species groups according to their current taxonomic boundaries (Fig. 3). In addition, this NJ network approach clustered most of the species in agreement with the Bayesian reconstruction depicted in Fig. 2. The species from La Palma formed a cluster closely related to the species from El Hierro (*C. duranii*). Those inhabiting eastern Tenerife (*C. webbianus*, *C. tagananensis*, *C. anagensis*, *C. metlesicsii* and *C. cf. sp. nova*) and western Tenerife (*C. canariensis* and *C. burchardii*) were also segregated into regional clades already resolved in the Bayesian and NJ phylogenetic analyses. Neighbor-Net analysis also revealed some phylogenetic conflicts not identified in the tree building analyses. For example, the species from El Hierro (*C. duranii*) showed clear reticulation, with an intermediate position between La Palma cluster and a lineage grouping two species from La Gomera (*C. satarataensis* and *C. dariasii*). Finally, NN analyses suggested a degree of reticulated connection between *C. ghomerythus* (from La Gomera) and *C. massonianus* (from Madeira), but this potential relationship was not supported by any Bayesian approach.

### Bayesian clustering and spatial analyses of the genetic structure

Using the matrix of inter-population *F*_ST_ distances, and the matrix of geographical distances in kilometres, the Mantel test indicated a significant correlation between genetic and geographical distances (r = 0.258; *p*-value<0.05). Similar results were obtained if the Madeiran populations were excluded from the analysis (Mantel test r = 0.326, *p*-value<0.05).

**Figure 4:**
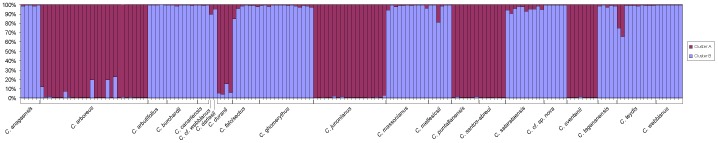
Results of STRUCTURE analyses of the entire AFLP dataset with *K* = 2. Bayesian estimation of genetic structure within Macaronesian *Cheirolophus* according to the best model proposed by STRUCTURE (*K* = 2).

The Bayesian analysis of population genetic structure conducted with STRUCTURE found the highest L(*K*) and Δ*K* values for *K* = 2. This grouping separated La Palma species (plus *C. duranii* from El Hierro) – cluster A – from the rest of the species from Tenerife, La Gomera, Gran Canaria and Madeira (including the *C. teydis* population from La Palma) – cluster B –, showing high percentages of individual memberships (97% and 98%, respectively) for these predefined groups (Fig. 4). According to the STRUCTURE analyses, the population of *C. teydis* from La Palma, some individuals of *C. arboreus*, *C. duranii* from El Hierro and one specimen of *C. metlesicsii* were the only ones presenting considerable (>10%) levels of admixture among the two genetic groups detected (between 14.9% and 34.3%). BAPS results showed a more fragmented distribution, with *K* = 11 as the most plausible number of clusters (P = 0.995; Figs. 1 and 3). The mixture analyses with or without spatially informative priors resulted in congruent assignment of individuals and no individual reassignment between the populations was observed. The genetic structure revealed by BAPS proved to be highly congruent with the phylogenetic analyses carried out (Fig. 3), showing at the same time a clear geographical pattern. Except for the populations of *C. teydis* and *C. massonianus*, the occurrence of genetic clusters determined by BAPS appeared to be limited to single islands and, in most cases, even restricted to particular regions within the islands (Fig. 1). No admixed individuals were detected according to this Bayesian clustering approach.

The results of non-hierarchical AMOVA indicated a highly significant level of genetic structure among populations (56.79%; df = 29; *p*-value<0.01, Table 1). The hierarchical AMOVA analyses also showed significant genetic differentiation explained at all levels and for all grouping schemes tested (all *p*-values<0.01, Table 1). The model that explained a larger fraction of variation among groups (38.63%) was the one considering the current taxonomic circumscription (*K* = 20). Using geographical origin (i.e. native island source, *K* = 7) as the grouping criterion revealed slightly lower, but also significant, genetic variance (23.59%) among sites. According to the structure proposed by the Bayesian clustering analyses, genetic differentiation among groups explained 18.73% of variation (*K* = 2; STRUCTURE) and 33.29% of variation (*K* = 11; BAPS).

**Table 1:** Analyses of molecular variance (AMOVA) of Macaronesian *Cheirolophus* species based on AFLP markers.

Source of variation	d.f.	Sum of squares	Variance components	Fixation indices	Percentage of variation	P
1. No population structure						
Among populations	28	2758.35	14.84	0.57	56.79	<0.0001
Within populations	143	1614.26	11.29		43.21	<0.0001
2. Species						
Among groups	19	2439.19	10.22	0.39	38.63	<0.0001
Among populations within groups	9	319.16	4.95	0.30	18.70	<0.0001
Within populations	143	1614.26	11.29	0.57	42.68	<0.0001
3. Islands						
Among groups	6	1236.50	6.55	0.24	23.59	<0.0001
Among populations within groups	22	1521.85	9.92	0.47	35.75	<0.0001
Within populations	143	1614.26	11.29	0.59	40.67	<0.0001
4. STRUCTURE						
Among groups	1	557.24	5.38	0.19	18.73	<0.0001
Among population within groups	27	2201.11	12.07	0.52	42.00	<0.0001
Within populations	143	161426	11.29	0.61	39.27	<0.0001
5. BAPS						
Among groups	10	1951.35	9.91	0.33	33.29	<0.0001
Among population within groups	18	807.00	6.58	0.37	24.55	<0.0001
Within populations	143	1614.26	11.29	0.58	42.15	<0.0001

The four possible scenarios considered were: no population structure (1), species delimitation (2), islands groups (3) STRUCTURE clustering (4) and BAPS clustering (5).

The PCoA using the first three principal coordinates explained 32.8% of the total variation in the data and confirmed several relationships detected in the phylogenetic and cluster analyses (Fig. S1). The first coordinate (accounting for 18.2% of the total variation) distinguished two main groups of species: the western Canarian taxa occurring on La Palma and El Hierro clearly segregated from another main cluster formed by species inhabiting Tenerife, Gran Canaria, La Gomera and Madeira. The second coordinate (representing 7.9% of variation) segregated *C. ghomerythus* populations in a different cluster, confirming the distinctiveness of this species from the other taxa on La Gomera. The addition of this second axis also separated *C. burchardii*, *C. canariensis* and *C. arbutifolius* accessions in one discrete cluster and *C. massonianus* individuals in another. Finally, plotting a third coordinate (accounting for 6.7% of the total variation) with the first axis, led to the distinction of four discrete clusters of species from: i) Tenerife and Gran Canaria; ii) La Gomera; iii) La Palma and El Hierro; and iv) Madeira.

### Morphological analyses and nrDNA sequencing data

The two first principal components represented 60% of the total morphological variation in the data set (Table S3). The first component (PC1) accounted for 31.4% of the total variance, showing the highest coefficients for flower colour and leaf shape traits (Table S3). PC2 represented another 28.4% of the total variance, having the highest coefficients for capitulum and leaf size and plant height traits. No correlation was found between genetic and morphological distances according to Mantel test (r = 0.00083, *p*-value>0.05), nor did any of the GAMOVA analysis show significant results for the morphological variables considered (all *p*-values>0.05).

The analysis of nrDNA sequences from [41] evidenced a considerable number of heteromorphic sites (double peaks) within potentially introgressed species. Table S2 shows nine positions (four observed in ITS regions and five in ETS region) presenting multiple peaks on the electropherograms in seven Macaronesian species.

## Discussion

### Species delimitation among Macaronesian *Cheirolophus*

The performance of diverse methods to analyse AFLP data sets has been found to be particularly important in evolutionary studies of plant radiations [45], [48], [62], [96]. Most of the analyses conducted in this study to evaluate the genetic structure of *Cheirolophus* in Macaronesia revealed different but complementary patterns, supporting the complex evolutionary history previously suggested for the genus in the archipelago [41]. Phylogenetic analyses based on AFLP were particularly useful in unravelling taxonomic boundaries among Macaronesian congeners. Both tree-building and network methods provided full support to the current species circumscription mainly based on morphological characteristics. Thus, our results corroborate the distinctiveness of these extraordinarily recent diverged species and support the utility of classical diagnostic characters employed in the taxonomical delimitation of Macaronesian *Cheirolophus*.

### Geographic differentiation and long-distance dispersal

AFLP analyses were not able to thoroughly disentangle the phylogeny of Macaronesian *Cheirolophus*, but assignment methods provided further evidence on the evolutionary relationship between different lineages. The model proposed by STRUCTURE (*K* = 2) identified a major geographical division, with the western islands (La Palma and El Hierro) clustering independently from the Central-Eastern ones (La Gomera, Tenerife, Gran Canaria), that grouped together with Madeira (Fig. 4). This genetic barrier has been also found in PCoA analyses (Fig. S1) and is supported by the phylogenetic signal (Figs. 2 and 3). Close evolutionary relationships among species from La Palma and El Hierro have already been reported in some studies concerning diverse animal and plant taxa from Macaronesia [97]–[99]. However, the majority of phylogeographic studies on Canarian flora and fauna have inferred that endemics from El Hierro appear to be more closely related to species from La Gomera than to those from La Palma [100]. Indeed, plastid DNA phylogeographic analysis has recently revealed a clear evolutionary connexion among El Hierro and La Gomera haplotypes [41], which provides evidence for a series of incongruent patterns among AFLP and cpDNA data (see discussion on introgression section below). A model with more clusters was proposed by BAPS (*K* = 11), suggesting as well significant influence of allopatric speciation in the evolutionary history of Macaronesian *Cheirolophus*. The species from Tenerife are grouped in three clusters corresponding to well differentiated regions of the island. One cluster comprised the taxa inhabiting mainly the eastern part of Tenerife (i.e. *C. webbianus*, *C. tagananensis*, *C. anagaensis*, *C. metlesicsii* and *C. cf. sp. nov.*). Another cluster groups *C. canariensis* and *C. burchardii* from the Teno mountains, at the western end of the island. This eastern/western pattern of geographic distribution in Tenerife has been recovered in other studies of several plant groups (see [101], for a review). In some cases, this segregation has been explained by disjunct evolution of lineages in two palaeo-islands of Tenerife [102], [103], currently corresponding to Anaga and Teno massifs at different ends of the island. According to previous phylogeographic and dating analyses [41], this geographic splitting of *Cheirolophus* lineages from eastern and western Tenerife could be related to connection-isolation cycles caused by volcanic activity during the last 2 My [104]. A third cluster included the populations of *C. teydis* from Tenerife (Las Cañadas) and La Palma, all of them living around the subalpine zone (1800–2200 m) on both islands. The representatives from Gran Canaria were placed together by BAPS in a separate cluster. This result may support the recent hypothesis of a single colonisation event for this island and the subsequent diversification process giving rise to *C. arbutifolius* and *C. falcisectus* [41]. Our Neighbor-Net and PCoA analyses pointed to an evolutionary closeness among Gran Canaria and Tenerife lineages, but we were not able to reconstruct their accurate phylogeographic relationship. Nonetheless, in agreement to the BAPS results, the species from La Gomera are grouped in two different clusters showing a clear geographic distribution pattern: one including *C. ghomerythus* from the northern coast of the island and the other one embedding *C. satarataensis* and its former subspecies *C. dariasii*, from the south and south-west of La Gomera. Certain evolutionary closeness among the two lineages from La Gomera was detected in the PCoA analyses, but their monophyly could not be confirmed. BAPS method also differentiated four geographically-related clusters of species in La Palma: *C. junonianus* from the south of the island; *C. arboreus* from the north and the west; *C. santos-abreui*, *C. puntallanensis* and *C. sventenii* (A.Santos) G.Kunkel from the north and north-eastern part of La Palma; and *C. teydis* from the summits of this island (and from Las Cañadas in Tenerife). Finally, *C. duranii* from El Hierro and *C. massonianus* from Madeira grouped in two separate clusters, suggesting as well that geographic isolation has been involved in the radiation of the genus.

According to these results, sporadic long-distance dispersal (LDD) events have to be considered to explain the numerous intra and inter-island colonisations resulting in the current distribution of genetic and taxonomic diversity in Macaronesian *Cheirolophus*. Unfortunately, straightforward evidences of LDD are very difficult to obtain [105], and we have not been able to provide direct proofs of these events occurring on our group of study. However, several supporting evidences (discussed below) suggest that Macaronesian *Cheirolophus* might have the potential to undergo successful LDD events. Birds and lizards have been found to be involved in seed long- dispersal of multiple species in the Canary Islands [106], [107] and other oceanic archipelagos [108], [109]. In the Galapagos and Azores, finches (granivorous birds from family Fringillidae) have been reported as legitimate seed dispersers of dry-fruited plants – including Asteraceae – implicated in LDD events between islands [109]–[111]. The goldfinch (*Carduelis carduelis* L.) has been observed in the Canary Islands feeding on *Cheirolophus* seeds [112], which suggests that bird-mediated dispersal could also be an important LDD mechanism in Macaronesian species. The role of birds in LDD events in *Cheirolophus* was also pointed by Garnatje et al. [113], who inferred that seabirds could have mediated the re-colonisation event from the Balearic Islands towards the continent in *C. intybaceus* (Lam.) Dostál. Finally, viable seeds from another Centaureinae species have been recovered from lizard guts discarded by predatory birds in the Canarian archipelago [114], supporting this kind of secondary seed dispersal as a likely mechanism involved in the LDD of Macaronesian *Cheirolophus*. All these data suggest that *Cheirolophus* achenes may be able to be transported through long distances but, given the lack of strong direct evidences in our case study and the inherent stochasticity of LDD process [105], speculation about potential role of these or other animals as LDD vectors must be made with extreme caution.

Furthermore, the capacity for successful long distance colonisation in *Cheirolophus* could also be enhanced by certain intrinsic biological features showed by this group of plants. The genus presents a pseudo-self-compatible mating system [115], that may be able to originate a sexually reproducing population from a single propagule, carrying at the same time more genetic variation than a seed from an autogamous population [116]. In summary, the results of our AFLP analysis are consistent with a role of allopatric divergence in the radiation of Macaronesian *Cheirolophus*; a hypothesis apparently supported by additional bibliographic evidences suggesting a certain degree of ability for successful long-distance colonisation events in the genus.

### Geographic-genetic correlation and limited gene flow in Macaronesian *Cheirolophus*

Genetic isolation has been proposed as a major factor determining plant speciation on oceanic islands [117]. The significance found in the spatial explicit analysis (Mantel test) suggests that Macaronesian *Cheirolophus* are influenced by geographic-genetic correlation across species. In particular, our results indicate that the more closely evolutionary-related species are as well geographically closer to each other, which is in good agreement with a gene flow scenario dominated by short distance events [118]. Regular seed dispersal in *Cheirolophus* has been reported to be limited to very short distances (see [32] for some species description). This genus shows relatively heavy and pappus-lacking cypselas that are unable to be transported by the wind, falling by gravity beside the mother plant during dissemination [119]. Another important factor enhancing geographic-genetic correlation across species might be topographic isolation [118]. Most *Cheirolophus* taxa inhabit deep ravines, coastal cliffs or steep slopes [30], [32], additionally preventing regular seed dispersal through long distances. The large number of Macaronesian *Cheirolophus* species inhabiting a few restricted and isolated locations (e.g. microendemisms such as *C. anagaensis*, *C. burchardii*, *C. dariasii*, *C. falcisectus, C. junonianus*, *C. metlesicsii, C. puntallanensis*, *C. santos-abreui* or *C. tagananensis*) could be related to the additive effect of poorly connected habitats with the limited seed dispersal capacity of *Cheirolophus*.

Our analysis across the whole distribution range of Macaronesian *Cheirolophus* revealed overall low levels of genetic diversity. This result is in accordance with the general expectation that endemic species, and particularly island endemics [120], exhibit lower levels of genetic diversity than widespread species [121], [122]. Generally, microendemics occupying restricted and isolated populations (e.g. *C. anagaensis*, *C. duranii*, *C. junonianus*, *C. massonianus*, *C. cf. sp. nova*, or *C. tagananensis*) show lower genetic diversity levels than species with numerous, widely distributed populations (e.g. *C. arboreus*, *C. ghomerythus*, *C. sventenii*, or *C. teydis*). However, this expected pattern is contradicted by certain populations of widely distributed species (e.g. *C. arbutifolius*, *C. satarataensis*) also showing lower genetic diversity values. Considering the limited number of sampled populations per species and the low number of individuals available, these indices could be underestimating genetic diversity, particularly in these widespread species. We found only three populations showing private fragments, probably due to the low sampling size within some populations and the strict choice of polymorphic bands.

Some of the genetic diversity indices studied in the Macaronesian *Cheirolophus* can also be compared with the values reported by Garnatje et al. [113] in the Mediterranean complex of *C. intybaceus*, which includes some taxa distributed in the eastern Iberian Peninsula and the Balearic Islands. Diversification in the *C. intybaceus* complex presumably started in the same period as the Macaronesian radiation [41], but in the Mediterranean group it resulted in only four doubtful species. Heterozygosity levels detected in populations of Macaronesian species are significantly lower than in Mediterranean populations of the *C. intybaceus* complex. Lower genetic variation has been associated with species showing limited geographical distribution, smaller populations and exhibiting little gene flow [123]–[125]. Our results could be reflecting that Macaronesian *Cheirolophus* – distributed in small populations across islands with steeply dissected topography – have been comparatively more isolated than Mediterranean ones, thus contributing to their progressive genetic divergence. As it has been proposed for other plant groups by Ellis et al. [118] and Knope et al. [22], the combination of certain ability for sporadic long-distance colonisation – see the section above – and poor gene flow capacity – due to both intrinsic and geographic characteristics – could have played an important role enhancing the explosive diversification of *Cheirolophus* in Macaronesia.

### Ecological adaptation

Geographic isolation may have been important for enhancing diversification but ecological adaptation is as well a common mechanism contributing – either in allopatry [126] or in sympatry [127] – to island plant speciation. According to Whittaker and Fernández-Palacios [1], the diversification of *Cheirolophus* in the Macaronesian archipelagos can be considered an example of non-adaptive radiation occurred on oceanic islands. Indeed, most species of this group exploit very similar niches in different islands, showing minor morphological differences. However, Macaronesian *Cheirolophus* present as well a few cases of taxa that have apparently adapted their morphology to the significantly diverse ecological conditions found on these archipelagos. *Cheirolophus teydis* is the only species of the genus that occupies the subalpine habitat, showing morphological adaptations to tougher ecological conditions (i.e. rosette-like disposed leaves with reduced laminas; waxy leaf cover; high number of smaller flowers; annual flowering shoots). The species from Gran Canaria – *C. arbutifolius* and *C. falcisectus* – are the result of a diversification process originated after a single colonisation of the island (see above). These species have diverged into different niches allopatrically within the same island – *C. falcisectus* inhabits more xeric habitats and shows clear leaf reduction while *C. arbutifolius* occupies more humid locations and present an arborescent habit and a larger leaf surface – thus suggesting an additional example of ecological differentiation. A similar case of ecological adaptation can be found in La Palma, where *C. junonianus* – from the south of the island and inhabiting significantly more arid localities than the rest of species from the northern part of this island – shows parallel morphological adaptation in size and leaf shape to drought conditions. Equivalent eco-morphological responses have already been reported in other Macaronesian plant taxa that have undergone an adaptive radiation process (e.g. *Argyranthemum* [2]). Interestingly, our morphology-genetics correlation analyses indicate that morphological similarity across the different Macaronesian *Cheirolophus* species is not affected by genetic similarity. Certainly, phylogenetic and clustering analyses are not congruent with a relationship between the main lineages and ecology: some species occupying different niches share the same or close genetic groups/lineages (e.g. *C. falcisectus* and *C. arbutifolius*; or *C. junonianus* and *C. arboreus*), whilst species showing similar habitats and morphological features frequently belong to very different genetic clusters/lineages (e.g. *C. duranii* and *C. tagananesis*; or *C. ghomerythus* and *C. burchardii*). These patterns suggest that the few cases of ecological adaptation (cited above) do not seem to be the result of a single eco-morphologic shift occurring at initial stages of the Macaronesian *Cheirolophus* diversification, but they may correspond to more recent, multiple and independent phenotype-environment differentiation processes.

Clearly, there are not enough data here to discard a vital role of selection in the radiation of *Cheirolophus*. We only measured a few morphological traits usually employed to delimitate taxonomically the Macaronesian species (Table S3), but other potentially important morphological and physiological features could have been missed. In addition, there is no accurate evaluation of the niches occupied by the different species, so there could also be fine-scale ecological variables differentiating habitats formerly considered as equivalent. Therefore, more precise inferences about the relative importance of ecological adaptation in this radiation process would require additional intraspecific sampling, supplementary morphological, physiological and ecological measurements as well as more appropriate tests (see [126], [128], [129]). Further studies applying these methodologies will improve our understanding of the role played by selection versus neutral differentiation in islands diversifications.

### Evidence of interspecific gene flow

Introgression is another mechanism formerly proposed to have played a role in the evolutionary history of Macaronesian *Cheirolophus* [41]. For instance, the species from Madeira, *C. massonianus*, was proposed to have come into contact with a continental congener, resulting in a chloroplast capture event. The genetic imprint of this hybridization event was not detected in the nrDNA regions sequenced in that former study, grouping *C. massonianus* within the Canarian clade and suggesting that introgression – via plastid transfer (see [130]) – did not affect the nuclear genome. From our AFLP data, pair-wise *F*_ST_ comparisons between Madeiran and Canarian populations showed similar genetic differentiation values to comparisons within Canarian populations (Table S4), apparently supporting the overall closeness among nuclear genomes of species from both archipelagos. Unfortunately, we did not include in this AFLP study any continental species putatively involved in *C. massonianus* hybridization, so our analyses do not allow assessment of whether introgression was limited to the chloroplast genome or also affected the nuclear genome.

In contrast, traces of genetic admixture were detected from AFLP data by STRUCTURE analysis in a population of *C. teydis* from La Palma (Fig. 4). Our results suggest that *C. teydis* originated in Tenerife and colonised subsequently La Palma, where genetic contact with other species from this island may occur. Plastid DNA analyses [41] support this hypothesis, pointing to a process of plastid capture to explain haplotypic differentiation among *C. teydis* populations from Tenerife and La Palma. In the same way, few *C. arboreus* individuals from populations in North-West La Palma show some genetic introgression among both genetic clusters defined by STRUCTURE. It has been reported that some specimens of *C. arboreus* from this part of the island exhibit morphological traits significantly distinct from the type [32]. Indeed, one of these *C. arboreus* populations from NW La Palma (Bco. Briestas) shows a different cpDNA haplotype from the rest of populations [41]. Another potential case of introgression could be affecting the species from El Hierro, *C. duranii*. This species is grouped together with the rest of taxa from La Palma according to our phylogenetic, PCoA and clustering analyses (Figs. 2, 4 and S1). In contrast, plastid DNA phylogenetic analysis [41], revealed a clear evolutionary closeness among *C. duranii* and the species from La Gomera. The Neighbor-Net analysis of our AFLP data mainly supports the evolutionary relationship among *C. duranii* from El Hierro and the rest of species from La Palma, but it also shows a faint reticulation signal between *C. duranii* and *C. satarataensis* from La Gomera (Fig. 3). In this case, certain genetic admixture – albeit very weak – can also be perceived from the STRUCTURE analysis, suggesting as well that this species from El Hierro may present genetic traces from both La Palma and La Gomera *Cheirolophus* species. Finally, the occurrence of introgression events during the radiation process could be also supported by the polymorphic sites observed in nrDNA sequences of the species here mentioned (see Table S2).

These results suggest that introgression might have played certain role in the evolutionary history of *Cheirolophus* in Macaronesia. However, similar patterns can be generated by ancestral polymorphisms and incomplete lineage sorting [131]. The STRUCTURE analysis does provide indication of admixed genotypes in some species, but at *K* = 2 the parent species for introgression are impossible to determine, being perhaps a vestige from some ancestral polymorphisms. According to the phylogeographic analysis performed by Vitales et al. [41], La Palma and El Hierro were colonised by *Cheirolophus* from Tenerife Island, so the genetic cluster A – mainly found in La Palma and El Hierro – proposed by STRUCTURE should be derived from the putative older cluster B, predominant in Tenerife, La Gomera and Gran Canaria (see Fig. 4). The admixture signal observed in *C. duranii* or in NW *C. arboreus* populations fits well with this alternative scenario considering certain retention of ancestral polymorphisms. In contrast, the pattern observed in *C. teydis* from La Palma – showing considerable admixture signal from the derived cluster B – seems more difficult to explain by the only action of ancient polymorphism retention. Heteromorphic positions found in nrDNA are consistent with some genetic reticulation but they can also be attributed to retention of polymorphisms and incomplete concerted evolution [132], especially considering the rapidity of this radiation process. In summary, there are some evidence for genetic introgression during the diversification of Macaronesian *Cheirolophus*, but in most cases there are alternative possible explanations (e.g. ancestral polymorphisms), thus limiting our conclusions about the relative importance of reticulation in the evolutionary history of the group.

### Conservation recommendations

The genus *Cheirolophus* is illustrating one of the largest plant radiations in the Canary Islands [133]. Having nine of the 20 extant insular species included in the IUCN Red List [31], *Cheirolophus* shows the highest proportion of endangered taxa for any species-rich lineage in this archipelago. Thus, attending to the major conservational interest of the group, we consider capital to analyse the results of this study from a conservation point of view. Even though Macaronesian *Cheirolophus* are the result of an exceptionally recent diversification, phylogenetic analyses based on AFLP confirmed the evolutionary identity of currently described endemics. Generally, the genetic diversity indexes calculated for the different populations and species of the group were found to be relatively heterogeneous, reflecting the complexity of this radiation process (see above). However, the frequency-down-weighted fragment values per population (DW) provided interesting and useful information about conservation biology of the numerous endangered representatives of the genus. This rarity index (DW), has been employed to assess the genetic distinctiveness of populations and species [134], and can also be used as an indicator of uniqueness and evolutionary relevance for conservation. Previous works suggest that species with high levels of unique genetic information are more likely to be threatened (e.g. [135], [136]). However, according to our results, current threat categories assigned to Macaronesian *Cheirolophus* species do not reflect their uneven evolutionary differentiation. *Cheirolophus* taxa considered in higher extinction risk categories (CR and EN) in the Spanish Red List of Endangered Flora [32] and IUCN Red List of Threatened Species [31] have relatively low DW values (e.g. *C. arboreus*, *C. duranii*, *C. metlesicsii*, *C. santos-abreui*). In contrast, those species showing higher DW values - therefore considered more genetically distinct – are assigned to lower extinction risk categories (VU) or even considered unthreatened (e.g. *C. canariensis*, *C. satarataensis*, *C. webbianus*). As resources for conservation are limited, their optimal allocation is essential [137]. Prioritization may be especially important when dealing with rich groups of closely related endemics inhabiting biodiversity hotspots [138]–[140], as is the case of Macaronesian *Cheirolophus*. We suggest that future evaluations of the endangered status of Macaronesian *Cheirolophus* should take into account the evolutionary distinctiveness results presented here. Predictably, the prioritization in resources allocation among *Cheirolophus* species would change if their genetic differentiation level is considered during conservation assessment process.

## Supporting Information

Figure S1 **Principal coordinates (PCoA) plot of AFLP data for the Macaronesian** ***Cheirolophus*** **populations included in this study.** Different symbols correspond to different populations as shown in the legend in the right side. Some populations groups that are well-differentiated and/or mentioned in the text are circled and named.
(TIF)

Table S1 **Sampling information and genetic diversity indexes assessed.** Taxa, locality, code, number of sampled individuals (N), and genetic diversity indexes assessed by AFLP in 29 populations of *Cheirolophus* from Macaronesia. Genetic indices: number of private fragments (f_u_); heterozygosity (Hj); percentage of polymorphic loci for a standardised sample size of three (*PLP* 1%); band richness for a standardised sample size of three (*Br*); and frequency-down-weighted marker values index (DW).
(DOC)

Table S2 **Polymorphic positions in nrDNA sequencing of some Canarian** ***Cheirolophus*** **taxa.** Positions in ITS and ETS sequences of some Canarian *Cheirolophus* specimens where more than one base is represented in a single amplification product, seen as subequal multiple peaks on the electropherograms. Data of sequences from Vitales et al. (2014).
(DOC)

Table S3 **Data from diagnostic morphological characters of Canarian** ***Cheirolophus*** **species.** Character loadings in first two principal components for the analysis of *Cheirolophus* morphological data (high loadings are highlighted in boldface type).
(DOC)

Table S4 **Pair-wise** ***F*** **_ST_ distances based on AFLP data calculated among the Macaronesian** ***Cheirolophus*** **populations included in this study.**
(XLS)
