# Supplementary figures and images for: Key Processes for Cheirolophus (Asteraceae) Diversification on Oceanic Islands Inferred from AFLP Data
Source: PLoS One. 2014 Nov 20;9(11):e113207. doi: 10.1371/journal.pone.0113207 (PMC4239036; doi:10.1371/journal.pone.0113207)

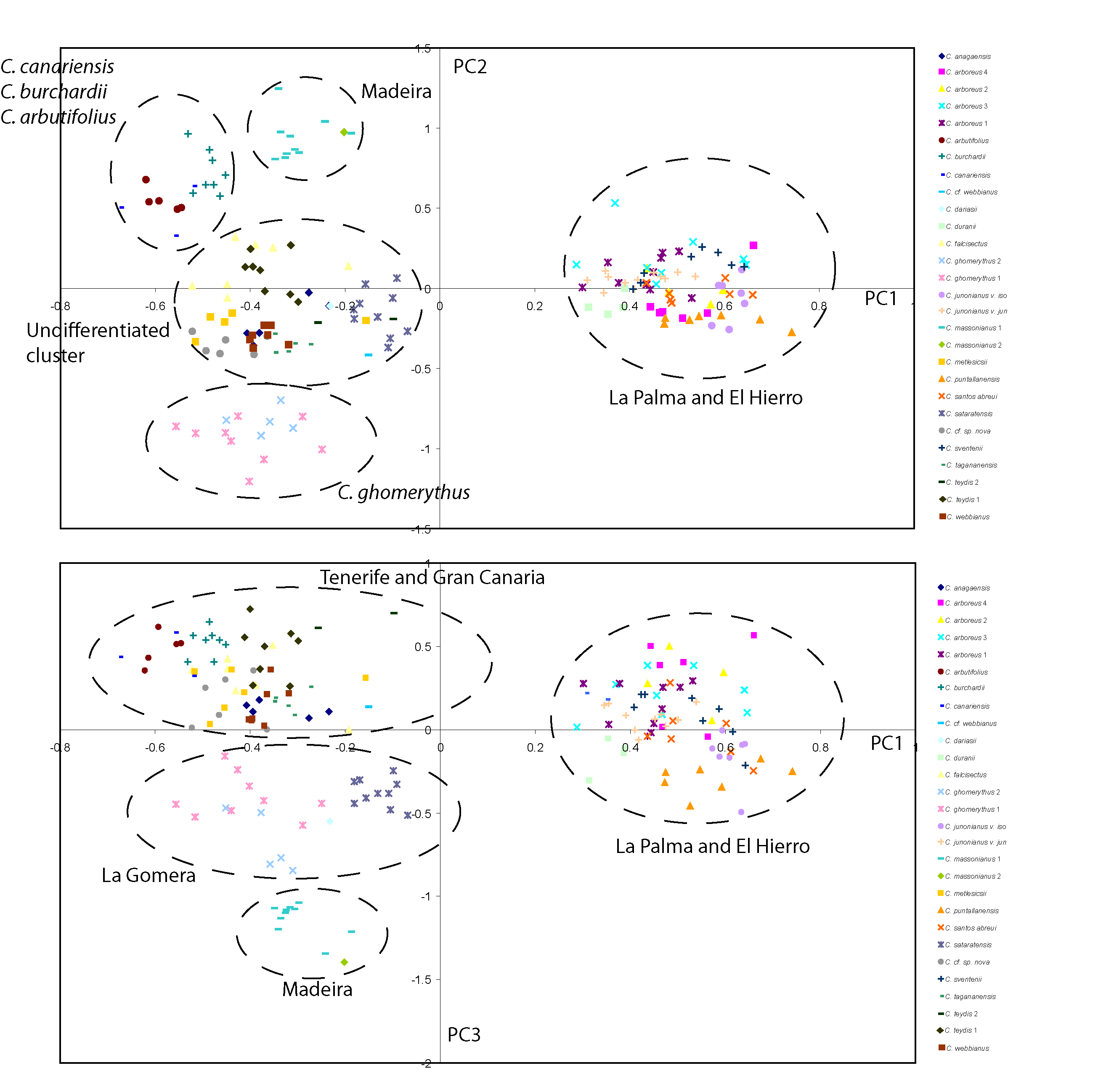

Supplement: Figure S1 — Principal coordinates (PCoA) plot of AFLP data for the Macaronesian Cheirolophus populations included in this study. Different symbols correspond to different populations as shown in the legend in the right side. Some populations groups that are well-differentiated and/or mentioned in the text are circled and named. (TIF) [file pone.0113207.s001.tif]
