# Supplementary material for: Key Processes for Cheirolophus (Asteraceae) Diversification on Oceanic Islands Inferred from AFLP Data
Source: PLoS One. 2014 Nov 20;9(11):e113207. doi: 10.1371/journal.pone.0113207 (PMC4239036; doi:10.1371/journal.pone.0113207)
Supplement: Table S1 — Sampling information and genetic diversity indexes assessed. Taxa, locality, code, number of sampled individuals (N), and genetic diversity indexes assessed by AFLP in 29 populations of Cheirolophus from Macaronesia. Genetic indices: number of private fragments (fu); heterozygosity (Hj); percentage of polymorphic loci for a standardised sample size of three (PLP 1%); band richness for a standardised sample size of three (Br); and frequency-down-weighted marker values index (DW). (DOC) [file pone.0113207.s002.doc]

**Table S1. Sampling information and genetic diversity indexes assessed.**

|  | **Locality** | **Cordinates** | **IUCN** | **N** | **f_u_** | **Hj** | ***PLP* (3)** | ***Br* (3)** | **DW (3)** |
| --- | --- | --- | --- | --- | --- | --- | --- | --- | --- |
| *Cheirolophus anagaensis* Santos | Spain: Tenerife, Anaga, Roque de los Pinos | -16.29 E  28.56 N | - | 5 | - | 0.0655 | 14.1 | 1.098 | 11.2133 |
| *Cheirolophus arboreus* (Webb & Berthel.) Holub 1 | Spain: La Palma, Barranco Briestas | -17.92 E  28.79 N | CR | 10 | - | 0.0967 | 26.2 | 1.137 | 11.2267 |
| *Cheirolophus arboreus* 2 | Spain: La Palma, La Cancelita | -17.89 E  28.68 N | CR | 4 | - | 0.1253 | 26.6 | 1.215 | 9.7587 |
| *Cheirolophus arboreus* 3 | Spain: La Palma, Barranco Fernando Porto | -17.92 E  28.81N | CR | 8 | - | 0.1039 | 29.0 | 1.160 | 10.7394 |
| *Cheirolophus arboreus* 4 | Spain: La Palma, Los Tilos | -17.79 E  28.79 N | CR | 6 | - | 0.1539 | 77.0 | 1.464 | 9.9710 |
| *Cheirolophus arbutifolius* (Svent.) G. Kunkel | Spain: Gran Canaria, Agaede | -15.65 E  28.07 N | VU | 5 | - | 0.0528 | 13.3 | 1.096 | 13.4243 |
| *Cheirolophus burchardii* Susanna | Spain: Tenerife, Buenavista, Teno | -16.88 E  28.36 N | VU | 8 | - | 0.1236 | 28.2 | 1.159 | 15.2535 |
| *Cheirolophus canariensis* (Brouss. ex Willd.) Holub | Spain: Tenerife, Los Carrizales | -16.86 E  28.31 N | VU | 3 | - | 0.0862 | 16.9 | 1.169 | 16.8559 |
| *Cheirolophus dariasii* (Svent.) Bramwell | Spain: Gomera, San Sebastian, El Cabrito | -17.17 E  28.09 N | CR | 1 | - | 0 | - | - | - |
| *Cheirolophus duranii* (Burchard) Holub | Spain: El Hierro, Temijiraque, Barranco Balcón | -17.93 E  27.77 N | CR | 4 | - | 0.0459 | 10.1 | 1.082 | 12.4477 |
| *Cheirolophus falcisectus* Montel. & Moral. | Spain: Gran Canaria, San Nicolás de Tolentino | -15.81 E  27.97 N | EN | 7 | - | 0.0987 | 23.8 | 1.145 | 12.4322 |
| *Cheirolophus ghomerythus* (Svent.) Holub 1 | Spain: La Gomera, Las Rosas | -17.21 E  28.17 N | EN | 9 | - | 0.1129 | 30.6 | 1.166 | 13.6423 |
| *Cheirolophus ghomerythus* 2 | Spain: La Gomery, La Tora | -17.23 E  28.19 N | EN | 5 | - | 0.1287 | 25.0 | 1.183 | 10.4128 |
| *Cheirolophus junonianus* (Svent.) Holub *v. isoplexiphyllus* G.Kunkel | Spain: La Palma, Fuencaliente, Las Machuqueras | -17.83 E  28.51 N | EN | 7 | - | 0.1009 | 21.4 | 1.129 | 8.6471 |
| *Cheirolophus junonianus* (Svent.) Holub *v. junonianus* | Spain: La Palma, Fuencaliente, Teneguía | -17.85 E  28.48 N | EN | 12 | - | 0.0714 | 17.3 | 1.080 | 10.0428 |
| *Cheirolophus massonianus* (Lowe) A. Hansen & Sunding 1 | Portugal: Madeira, Cabo Girao | -17.00 E  32.65 N | EN | 10 | - | 0.0989 | 25.0 | 1.121 | 10.7686 |
| *Cheirolophus massonianus* 2 | Portugal: Porto Santo, Pico Branco | -16.30 E  33.09 N | EN | 1 | - | 0 | - | - | - |
| *Cheirolophus metlesicsii* Montel 1 | Spain: Tenerife, Arafo, Barranco Añavingo 1 | -16.41 E  28.33 N | CR | 5 | - | 0.1003 | 22.6 | 1.163 | 11.3160 |
| *Cheirolophus metlesicsii* 2 | Spain: Tenerife, Arafo, Barranco Añavingo 2 | -16.43 E  28.35 N | CR | 1 | - | 0 | - | - | - |
| *Cheirolophus puntallanensis* Santos | Spain: La Palma, Puntallana, Barranco Nogales | -17.77 E  28.76 N | - | 7 | - | 0.0985 | 21.0 | 1.128 | 9.5019 |
| *Cheirolophus santos-abreui* Santos | Spain: La Palma, Barranco Madera | -17.8 E  28.71 N | CR | 7 | 1 | 0.0923 | 21.4 | 1.131 | 10.1761 |
| *Cheirolophus satarataensis* (Svent.) Holub | Spain: La Gomera, Alajeró | -17.29 E  28.1 N | VU | 10 | 1 | 0.0744 | 19.8 | 1.097 | 19.0523 |
| *Cheirolophus cf. sp. nova* | Spain: Tenerife, near Taganana, Afur. | -16.24 E  28.56 N | - | 6 | - | 0.0521 | 12.5 | 1.083 | 14.9196 |
| *Cheirolophus cf. webbianus* | Spain: Tenerife, Tagana, Barranco el Agua | -16.19 E  28.56 N | - | 1 | - | 0 | - | - | - |
| *Cheirolophus sventenii* (A. Santos) G. Kunkel | Spain: La Palma, Barlovento. | -17.85 E  28.81 N | VU | 8 | - | 0.1182 | 28.2 | 1.165 | 11.9495 |
| *Cheirolophus tagananesis* (Svent.) Holub | Spain:Tenerife, Taganana, Roque de las Ánimas | -16.21 E  28.56 N | VU | 5 | 1 | 0.0575 | 14.5 | 1.104 | 12.2485 |
| *Cheirolophus teydis* (Chr. P. Sm.) G. López 1 | Spain: Tenerife, Bocatauce | -16.68 E  28.21 N |  | 8 | - | 0.1033 | 26.6 | 1.156 | 14.5955 |
| *Cheirolophus teydis* 2 | Spain: La Palma, Garafía, Tagamantera | -17.86 E  28.77 N | - | 2 | - | 0.0572 | - | - | - |
| *Cheirolophus webbianus* (Sch.Bip.) Holub | Spain: Tenerife, Anaga, Chinamada | -16.28 E  28.57 N | VU | 7 | - | 0.0828 | 21.8 | 1.127 | 19.7497 |

Taxa, locality, decimal coordinates, IUCN threat category, number of sampled individuals (N), and genetic diversity indexes assessed by AFLP in 29 populations of *Cheirolphus* from Macaronesia. Genetic indices: Number of private fragments (f_u_); Heterozygosity (Hj); Percentage of polymorphic loci for a standardised sample size of three (*PLP* 1%); Band richness for a standardised sample size of three (*Br*); and frequency-down-weighted marker values index (DW).
