# Supplementary material for: Key Processes for Cheirolophus (Asteraceae) Diversification on Oceanic Islands Inferred from AFLP Data
Source: PLoS One. 2014 Nov 20;9(11):e113207. doi: 10.1371/journal.pone.0113207 (PMC4239036; doi:10.1371/journal.pone.0113207)
Supplement: Table S2 — Polymorphic positions in nrDNA sequencing of some Canarian Cheirolophus taxa. Positions in ITS and ETS sequences of some Canarian Cheirolophus specimens where more than one base is represented in a single amplification product, seen as subequal multiple peaks on the electropherograms. Data of sequences from Vitales et al. (2014). (DOC) [file pone.0113207.s003.doc]

Table S2. Positions in ITS and ETS sequences of some Canarian *Cheirolophus* specimens where more than one base is represented in a single amplification product, seen as subequal multiple peaks on the electropherograms. Data of sequences from Vitales et al. (2014).

| **Regions** | ***ITS*** | | | | ***ETS*** | | | | |
| --- | --- | --- | --- | --- | --- | --- | --- | --- | --- |
| Positions  Specimen | 164 | 207 | 223 | 677 | 165 | 229 | 307 | 587 | 589 |
| *C. arboreus* (Briestas) | G/A | G/T | G/A | T/C | T/C | G | T/C | T | T/C |
| *C. junonianus* var. *junonianus* | A | T | A | C | T/C | G | C | T | C |
| *C. sventenii* | G/A | G/T | G/A | C | T/C | G | C | T | T/C |
| *C. teydis* (LP) | G | T | A | T/C | C | G | C | A/T | C |
| *C. teydis* (T) | G | T | A | T | T/C | G | C | A | C |
| *C. duranii* | A | T | A | C | C | G/C | C | T | C |
| *C. canariensis* | G | T | A | T | C | G | C | A | C |
