# Supplementary material for: Key Processes for Cheirolophus (Asteraceae) Diversification on Oceanic Islands Inferred from AFLP Data
Source: PLoS One. 2014 Nov 20;9(11):e113207. doi: 10.1371/journal.pone.0113207 (PMC4239036; doi:10.1371/journal.pone.0113207)
Supplement: Table S3 — Data from diagnostic morphological characters of Canarian Cheirolophus species. Character loadings in first two principal components for the analysis of Cheirolophus morphological data (high loadings are highlighted in boldface type). (DOC) [file pone.0113207.s004.doc]

| **Species** | **Maximum height (cm)** | **Maximum leaf lenght (cm)** | **Maximum leaf width (cm)** | **Maximum capitulum diameter (cm)** | **Flower^1^colour** | **Leaf^2^ shape** |
| --- | --- | --- | --- | --- | --- | --- |
| *C. arboreus* | 350 | 8.7 | 2.8 | 2.5 | 1 | 1 |
| *C. arbutifolius* | 335 | 12 | 3.1 | 4.0 | -1 | 1 |
| *C. buchardii* | 150 | 12 | 1.6 | 1.5 | -1 | -1 |
| *C. canariensis* | 150 | 12.4 | 4.9 | 1.4 | -1 | -1 |
| *C. dariasii* | 60 | 15 | 4.0 | 4.0 | 1 | 1 |
| *C. duranii* | 100 | 8 | 3.0 | 1.5 | 1 | 1 |
| *C. falcisectus* | 118 | 15 | 5.0 | 1.8 | -1 | -1 |
| *C. ghomerythus* | 100 | 9.3 | 1.0 | 1.7 | -1 | 0 |
| *C. junonianus* | 70 | 10 | 3.0 | 1.4 | -1 | 0 |
| *C. metlesicsii* | 250 | 21 | 3.0 | 2.0 | -1 | 1 |
| *C. santos-abreui* | 200 | 13 | 3.0 | 2.0 | -1 | 1 |
| *C. satarataensis* | 150 | 15 | 4.0 | 1.5 | 1 | 1 |
| *C. tagananensis* | 100 | 13 | 4.0 | 1.8 | 1 | 1 |
| *C. cf. webbianus* | 138 | 11.2 | 2.8 | 1.9 | 1 | 1 |
| *C. teydis* | 150 | 5.3 | 0.7 | 1.6 | 1 | 1 |
| *C. webbianus* | 153 | 10.9 | 3.5 | 1.6 | 1 | 1 |
| **Components loadings** | |  |  |  |  |  |
| PC1 (31.4 %) | 0.2270 | -0.2745 | -0.3128 | 0.2714 | **0.5451** | **0.6360** |
| PC2 (28.4 %) | -0.3937 | **0.5651** | **-0.4320** | **-0.5388** | 0.1121 | -0.1900 |

Table S3. Data from diagnostic morphological characters of Canarian *Cheirolophus* species. Character loadings in first two principal components for the analysis of Cheirolophus morphological data (high loadings are highlighted in boldface type).

**^1^** “+1” for whitish and “-1” for rose-colored flowers.

**^2^** “+1” for entire, “-1” for divided and “0” for intermediate/both leaf shapes.
